# Characteristics of Adolescents and Young adults with HIV in the Republic of Korea from 2010 through 2015

**DOI:** 10.1038/s41598-020-66314-0

**Published:** 2020-06-10

**Authors:** Myeongsu Yoo, Jaehyun Seong, Jae-Gyu Yoon, Jeong-ok Cha, Yoon-Seok Chung, Kisoon Kim, Mee-Kyung Kee

**Affiliations:** 1 Division of Viral Disease Research, Center for Infectious Disease Research, Korea National Institute of Health, Chungbuk, Republic of Korea; 2 Division of TB and HIV/AIDS control, Center for Disease Prevention, Korea Center for Disease Control & Prevention, Chungbuk, Republic of Korea; 3 Division of Viral Disease, Center for Laboratory Control of Infectious Disease, Korea Center for Disease Control & Prevention, Chungbuk, Republic of Korea

**Keywords:** Diseases, Risk factors, Health care, Health policy, Public health

## Abstract

Although the number of newly diagnosed cases of HIV is decreasing worldwide, those in Korea have been steadily on the rise, especially among adolescents (ages 10–19 years) and young adults (ages 20–29 years). To identify the characteristics in the new diagnosis among these age groups, we analyzed HIV testing sizes and HIV prevalence under the national HIV surveillance system in Korea in the last six years. We collected data of HIV tests conducted at Blood Banks (BB), Public Health Centers (PHCs), and Military Manpower Administration (MMA) nationwide every year from 2010 to 2015, except for anonymous tests. HIV prevalence, calculated as the number of new HIV-diagnosed cases per 10,000 test-takers per year, was analyzed according to sex, age, institution, and reason for HIV testing. Data were analyzed using logistic regression. In the three testing institutes, there were new cases of HIV with 50% and 95% of cases diagnosed in young adults and adolescents, respectively. The total size of HIV tests at the three sites was approximately 3.5 million tests per year; 80% of these were conducted in BBs, 10% in PHCs, and 10% in MMA. HIV prevalence, according to age, increased across all age groups for the six years, especially prevalent in young adults doubled during that period (1.01 per 10,000 test-takers in 2010, 2.45 in 2015). HIV prevalence among the “suspected” young male adults who visited PHCs for tests, was highest during the six years, increasing 6.5 times in the last two years (315.79 per 10,000 test-takers in 2014, 335.55 in 2015) compared to before 2014. We identified the characteristics of growing HIV infection in Korea as the increase of HIV prevalence among the suspected of young male adults at PHCs. Further, we propose that HIV prevalence in MMA can be used as an essential index for national HIV surveillance of adolescent boys in Korea.

## Introduction

HIV infection, known to the public since the early 1980s, has been rapidly spreading worldwide through sexual contact, vertical transmission, blood transfusions, and needle sharing for injection drug use (IDU). The HIV epidemic has different transmission routes and the speed of spreading across countries^1^. Globally, for more than 35 years, approximately 78 million individuals have been infected with HIV, with 35 million HIV-related deaths, and 37 million people are currently living with HIV^2^. The first HIV-infection in Korea was identified in 1985, and over the next 30 years or so, approximately 15,000 individuals were identified as HIV-infected. It was reported that more than 99% of individuals diagnosed with HIV in Korea were infected via sexual contact, while transmission through blood transfusion, vertical transmission, and needle sharing for IDU was infrequent^3^. Soon after the discovery of HIV in Korea, the country initiated a prevention program focused on HIV transmission reduction by regularly performing HIV screening tests on high-risk groups for sexually transmitted infections (STI) and extending it to other groups^4–6^. Hospitals or clinics and public health centres (PHCs) have performed voluntary HIV screening, including health checkups and anonymous testing for suspected infection cases. Additionally, blood banks (BBs) and the Military Manpower Administration (MMA) have performed HIV screening for blood donors and men undergoing physical examination for conscription, respectively^7,8^. Thus far, Korea has emphasized on early diagnosis and active treatment of HIV to prevent its transmission^9^. As a consequence, Korea is known to have a low prevalence and slow spread of HIV infection when compared to other countries^10^.

For the last 40 years, numerous studies have been conducted to develop HIV therapies to eradicate HIV/AIDS among humans worldwide. However, there are obstacles to developing a preventative vaccine for HIV infection, and there is no cure. Hence, the disease burden of HIV/AIDS remains high. Accordingly, with the United Nations Program on HIV and AIDS (UNAIDS), each country has been implementing 90-90-90 and 95-95-95 strategies aiming to abolish the HIV epidemic by 2020 and 2030, respectively. Therefore, recently the global numbers of newly diagnosed HIV cases and HIV-related deaths have been decreasing gradually^11^.

In Korea, however, since 2013, the annual number of patients newly diagnosed with HIV has exceeded 1,000, and the age at diagnosis has been decreasing. Accordingly, this study was conducted to identify the characteristics of the increase of newly diagnosed HIV cases among adolescents and young adults in Korea. Specifically, the study examined the changes over the last six years in the sizes of HIV screening and the characteristics of HIV test-takers among blood donors, visitors to PHCs nationwide, and men undergoing health examination for conscription, most of whom tend to be young. Based on the findings, we aimed to analyze HIV prevalence and trends, as well as the characteristics of newly infected adolescents and young adults.

## Methods

### HIV testing system and data collection from HIV screening testing institute

Enzyme-linked immunosorbent assays, chemiluminescence immunoassays, fluorometric enzyme-linked immunoassays, and particle agglutination assays were used for HIV screening. PHCs may use a rapid HIV test kit if the screening is performed anonymously. If an HIV screening test is positive, the first testing institute refers the case to the Regional Institute of Health and Environment (IHE). The IHE then performs a western blot test for HIV and a confirmatory test for the final determination of HIV infection. If the western blot test is positive, the office reports the case to the Korea Centers for Disease Control and Prevention (KCDC), which registers individuals on the HIV database system and requests the corresponding PHC to perform an epidemiological investigation^9^. The study data that was collected visitors to PHCs under the National HIV Surveillance System in Korea was waived the requirement for written consent to participate.

KCDC collects annual data on HIV screening tests conducted at BBs, PHCs, and MMA. Specifically, the annual HIV screening data from approximately 250 PHCs nationwide included the following raw data: specimen number, sex, date of birth, screening date, nationality, the reason for testing, test results, the first referring institute, classification code, specimen number for confirmatory testing, and final test results^12,13^. The annual data provided by BBs included the number of HIV screening tests and the number of screened individuals by age, sex, occupation, and region. The annual data provided by MMA included an age-specific number of individuals screened for HIV.

### Participants

Study participants were the new HIV-diagnosed individuals in Korea and HIV test-takers in BBs, PHCs, and MMA between 2010 and 2015. Individuals previously diagnosed with HIV who had already registered in the HIV patient registry were excluded from the study so that we could analyze the annual trend in new HIV detections and change of HIV prevalence. Moreover, individuals who tested anonymously at PHCs were excluded from the study because epidemiological information, such as age, sex, and nationality, was not available. The participants whose data were collected from MMA were males of Korean nationality undergoing physical examinations for compulsory military service qualification determination; this information was mandatory for all males of Korean nationality under the age of 20^8^.

### Data statistical analysis

All individuals undergoing physical examination at the MMA were male, while the blood donors and PHC visitors were male and female. HIV-infected patients were classified into six age groups: <20, 20–29, 30–39, 40–49, 50–59, and ≥60 years. HIV test-takers were divided into three age (years) groups: <20 (adolescents), 20–29 (young adults), and ≥30 (adults). The reason for HIV testing in blood donors and male undergoing physical examination for conscription was classified as blood donation and conscription, respectively. For PHC visitors, the reasons for HIV testing were diverse. They were classified with the same classification scheme used in a previous study^12^ as follows: health check-up, medical certificate, prenatal check-up, referral by doctor, suspected for HIV infection, patient with tuberculosis, prisoner, and STI risk group. Annual HIV prevalence was defined as the number of confirmed HIV-positive individuals per 10,000 HIV test-takers in 1 year. Those previously identified as HIV-positive were excluded from the counts of HIV test takers and those identified as HIV-positive. Statistical analysis was performed using SAS 9.4. Logistic regression analysis was conducted to examine the difference in prevalence for each variable. Statistical significance was set at 0.05 for all cases. This study was obtained approval for study design from the KCDC Institutional Review Board (approval no. 2016-07-06-PE-A). Also, the informed consents from study participants were waived the requirement for written by the KCDC Institutional Review Board.

## Results

### The status and trends of HIV infection by age and testing institute

**Figure 1:**
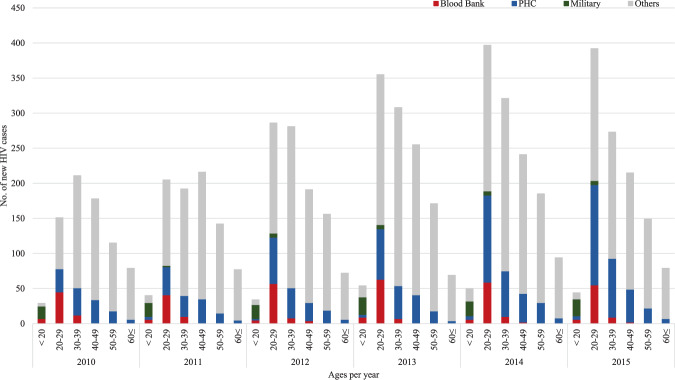
Distribution of newly diagnosed HIV-positive patients by age and type of HIV screening institution in which the initial diagnosis was performed, in Korea, from 2010 to 2015; PHC: public health centers, Military: the Military Manpower Administration, Others: hospitals or clinics etc.

In Korea, 837 individuals were identified as HIV-infected in 2010, and the figure has exceeded 1,000 since 2013, increasing to 1,191 and 1,152 in 2014 and 2015, respectively. The number of HIV-infected individuals identified in BBs, PHCs, and MMA was 212 in 2010, and the number almost doubled to 410 in 2015. Focusing on adolescents and young adults, the number of HIV-infected individuals was 103 in 2010 and 239 in 2015, an increase of more than twice in 6 years. These figures are approximately 50% of the total annual number of HIV infections in adolescents and young adults at a national level, and the proportion did not change year to year. Adolescents aged 10–19 years identified at the 3 institutes constituted 96% of the total number of HIV-infected individuals in the same age range; young adults aged 20–29 years constituted more than 50% of the total number of HIV infections in the corresponding age range. According to testing institutes, HIV-infected individuals identified through a physical examination at MMA accounted for approximately 50% of the total number of HIV-infected individuals in the 10–19-year age group. The proportions were approximately 18% and 28% in the case of blood donors and PHC visitors, respectively [Fig. 1].

### The sizes of HIV tests and the characteristics in HIV prevalence in three HIV screening institutions

The sizes of HIV tests administered in BBs, PHCs, and MMA and the characteristics of HIV prevalence between 2010 and 2015 were identified and analyzed and identified [Table 1]. The number of HIV tests conducted at the three testing institutes increased from approximately 3.2 million in 2010 to 3.6 million in 2015. Annually, BBs performed 2.5–2.9 million tests, which included 1.7 million test-takers. The annual number of tests for PHCs nationwide and the MMA were 360,000–420,000 tests (approximately 350,000 individuals) and 350,000–380,000 tests (or individuals), respectively. Thus, the number of HIV tests was similar between PHCs and the MMA, and the number of tests at BBs was almost 7-times higher than those at PHCs and the MMA. In 2015, the number of HIV tests conducted at BBs increased by 350,000 compared to 2010, but the number of test-takers did not increase (annual rate of repeat tests per blood donor was 1.47 in 2010 and 1.67 in 2015).

**Table 1:** Trend in the number of HIV screening tests and characteristics of HIV prevalence at three HIV testing institutions in Korea from 2010 to 2015.

	Category		2010			2011			2012			2013			2014			2015		
			Tests (N) (%)	Persons(N) (HIV+)	Pre. (95% CI)	Tests (N) (%)	Persons(N) (HIV+)	Pre. (95% CI)	Tests (N) (%)	Persons(N) (HIV+)	Pre. (95% CI)	Tests (N) (%)	Persons(N) (HIV+)	Pre. (95% CI)	Tests (N) (%)	Persons(N) (HIV+)	Pre. (95% CI)	Tests (N) (%)	Persons(N) (HIV+)	Pre. (95% CI)
Total	Total		3,240,999	2,390,380	0.89	3,205,492	2,343,128	0.89	3,325,958	2,431,414	1.08	3,510,202	2,481,035	1.19	3,614,877	2,458,283	1.53	3,630,867	2,436,248	1.68
				(212)	(0.77–1.01)		(208)	(0.77–1.01)		(263)	(0.95–1.21)		(296)	(1.06–1.33)		(377)	(1.38–1.69)		(410)	(1.52–1.85)
	**Sex**																			
		Male	2,271,861	1,674,098	1.19	2,210,502	1,610,374	1.20	2,295,383	1,657,410	1.49	2,419,557	1,689,491	1.53	2,490,248	1,661,759	2.13	2,494,041	1,636,773	2.33
			(70.1)	(200)	(1.03–1.36)	(69.0)	(194)	(1.04–1.37)	(69.0)	(247)	(1.30–1.68)	(68.9)	(259)	(1.35–1.72)	(68.9)	(354)	(1.91–2.35)	(68.7)	(382)	(2.10–2.57)
		Female	969,138	716,282	0.17	994,990	732,754	0.19	1,030,575	774,004	0.21	1,090,645	791,544	0.47	1,124,629	796,524	0.29	1,136,826	799,475	0.35
			(29.9)	(12)	(0.07–0.26)	(31.0)	(14)	(0.09–0.29)	(31.0)	(16)	(0.11–0.31)	(31.1)	(37)	(0.32–0.62)	(31.1)	(23)	(0.17–0.41)	(31.3)	(28)	(0.22–0.48)
	**Age**																			
		10–19	1,350,772	1,099,858	0.23	1,395,076	1,127,882	0.27	1,355,146	1,100,068	0.25	1,368,084	1,094,803	0.35	1,376,586	1,078,621	0.30	1,339,385	1,035,618	0.34
			(41.7)	(25)	(0.14–0.32)	(43.5)	(30)	(0.17–0.36)	(40.7)	(27)	(0.15–0.34)	(39.0)	(38)	(0.24–0.46)	(38.1)	(32)	(0.19–0.40)	(36.9)	(35)	(0.23–0.45)
		20–29	1,152,858	771,086	1.01	1,083,316	728,909	1.14	1,131,485	753,531	1.71	1,268,308	814,429	1.73	1,339,860	826,116	2.29	1,353,018	832,324	2.45
			(35.6)	(78)	(0.79–1.24)	(33.8)	(83)	(0.89–1.38)	(34.0)	(129)	(1.42–2.01)	(36.1)	(141)	(1.45–2.02)	(37.1)	(189)	(1.96–2.61)	(37.3)	(204)	(2.11–2.79)
		30≤	737,369	519,436	2.10	727,100	486,337	1.95	839,327	577,815	1.85	873,810	571,803	2.15	898,431	553,546	2.82	938,464	568,306	3.01
			(22.8)	(109)	(1.70–2.49)	(22.7)	(95)	(1.56–2.35)	(25.9)	(107)	(1.50–2.20)	(24.9)	(123)	(1.77–2.53)	(24.9)	(156)	(2.38–3.26)	(25.8)	(171)	(2.56–3.46)
BB	Total	2,514,699	1,716,398	0.37	2,448,516	1,638,553	0.35	2,542,495	1,683,383	0.45	2,708,173	1,722,200	0.46	2,844,538	1,730,644	0.45	2,864,301	1,710,422	0.43	
				(64)	(0.28–0.46)		(57)	(0.26–0.44)		(75)	(0.34–0.55)		(80)	(0.36–0.57)		(78)	(0.35–0.55)		(73)	(0.33–0.52)
	**Sex**																			
		Male	1,797,670	1,208,277	0.52	1,717,256	1,126,991	0.50	1,790,590	1,159,518	0.63	1,909,293	1,186,075	0.66	2,007,168	1,186,064	0.62	2,024,048	1,173,234	0.61
			(71.5)	(63)	(0.39–0.46)	(70.1)	(56)	(0.37–0.63)	(70.4)	(73)	(0.49–0.77)	(70.5)	(78)	(0.51–0.80)	(70.6)	(73)	(0.47–0.76)	(70.7)	(71)	(0.46–0.75)
		Female	717,029	508,121	0.02	731,260	511,562	0.02	751,905	523,865	0.04	798,880	536,125	0.04	837,370	544,580	0.09	840,253	537,188	0.04
			(28.5)	(1)	(0.0–0.06)	(29.9)	(1)	(0.0–0.06)	(29.6)	(2)	(0.00–0.09)	(29.5)	(2)	(0.00–0.09)	(29.4)	(5)	(0.01–0.17)	(29.3)	(2)	(0.00–0.09)
	**Age**																			
		10–19	974,759	729,101	0.10	993,913	731,145	0.08	979,285	725,236	0.07	981,978	711,624	0.13	997,296	701,880	0.09	974,027	673,127	0.09
			(38.8)	(7)	(0.02–0.17)	(40.6)	(6)	(0.02–0.15)	(38.5)	(5)	(0.01–0.13)	(36.3)	(9)	(0.04–0.21)	(35.1)	(6)	(0.02–0.15)	(34.0)	(6)	(0.02–0.16)
		20–29	1,028,581	671,905	0.67	955,789	617,605	0.66	1,023,235	652,522	0.87	1,147,317	701,799	0.90	1,223,228	717,878	0.82	1,234,936	721,058	0.76
			(40.9)	(45)	(0.47–0.87)	(39.0)	(41)	(0.46–0.87)	(40.2)	(57)	(0.65–1.10)	(42.4)	(63)	(0.68–1.12)	(43.0)	(59)	(0.61–1.03)	(43.1)	(55)	(0.56–0.96)
		30≤	511,359	315,392	0.38	498,814	289,803	0.35	539,975	305,625	0.43	578,878	308,777	0.45	624,014	310,886	0.42	655,338	316,237	0.38
			(20.3)	(12)	(0.17–0.60)	(20.4)	(10)	(0.13–0.56)	(21.2)	(13)	(0.19–0.66)	(21.4)	(14)	(0.22–0.69)	(21.9)	(13)	(0.19–0.65)	(22.9)	(12)	(0.16–0.59)
PHCs	Total	369,926	317,608	4.09	385,985	333,584	3.87	411,080	375,648	4.31	427,406	384,212	4.82	396,507	353,807	7.69	403,642	362,902	8.46	
				(130)	(3.39–4.80)		(129)	(3.20–4.53)		(162)	(3.65–4.98)		(185)	(4.12–5.51)		(272)	(6.77–8.60)		(307)	(7.51–9.41)
	**Sex**																			
		Male	117,817	109,447	10.87	122,255	112,392	10.32	132,410	125,509	11.79	135,641	128,793	11.65	109,248	101,863	24.94	107,069	100,615	27.93
			(31.8)	(119)	(8.92–12.83)	(31.7)	(116)	(8.4–12.2)	(32.2)	(148)	(9.89–13.69)	(31.7)	(150)	(9.78–13.51)	(27.6)	(257)	(21.87–28.00)	(26.5)	(281)	(24.67–31.19)
		Female	252,109	208,161	0.53	263,730	221,192	0.59	278,670	250,139	0.56	291,765	255,419	1.37	287,259	251,944	0.71	296,573	262,287	0.99
			(68.2)	(11)	(0.22–0.84)	(68.3)	(13)	(0.27–0.91)	(67.8)	(14)	(0.27–0.85)	(68.3)	(35)	(0.92–1.82)	(72.4)	(18)	(0.38–1.04)	(73.5)	(26)	(0.61–1.37)
	**Age**																			
		10–19	25,080	19,824	0.00	33,391	28,965	1.38	17,654	16,625	1.20	26,818	23,891	1.67	21,808	19,259	2.60	20,151	17,284	2.89
			(6.8)	(0)	(0.00–0.00)	(8.7)	(4)	(0.03–2.73)	(4.3)	(2)	(0.0–2.87)	(6.3)	(4)	(0.03–3.31)	(5.5)	(5)	(0.32–4.87)	(5.0)	(5)	(0.36–5.43)
		20–29	118,894	93,798	3.52	124,334	108,111	3.70	94,124	86,883	7.60	105,709	97,348	7.40	100,336	91,942	13.49	100,432	93,616	15.28
			(32.1)	(33)	(2.32–4.72)	(32.2)	(40)	(2.55–4.85)	(22.9)	(66)	(5.76–9.43)	(24.7)	(72)	(5.69–9.10)	(25.3)	(124)	(11.11–15.86)	(24.9)	(143)	(12.77–17.78)
		30≤	222.952	203,986	4.76	228,260	196,508	4.33	299,302	272,140	3.45	294,879	262,973	4.14	274,363	242,606	5.89	283,059	252,002	6.31
			(61.1)	(97)	(3.81–5.70)	(59.1)	(85)	(3.41–5.24)	(72.8)	(94)	(2.76–4.15)	(69.0)	(109)	(3.37–4.92)	(69.2)	(143)	(4.93–6.86)	(70.1)	(159)	(5.33–7.29)
MMA	Total	356,374	356,374	0.51	370,991	370,991	0.59	372,383	372,383	0.70	374,623	374,623	0.83	373,832	373,832	0.72	362,924	362,924	0.83	
				(18)	(0.27–0.74)		(22)	(0.35–0.84)		(26)	(0.43–0.97)		(31)	(0.54–1.12)		(27)	(0.45–0.99)		(30)	(0.53–1.12)
	**Age**																			
		10–19	350,933	350,933	0.51	367,772	367,772	0.54	358,207	358,207	0.56	359,288	359,288	0.70	357,482	357,482	0.59	345,207	345,207	0.70
			(98.5)	(18)	(0.28–0.75)	(99.1)	(20)	(0.31–0.78)	(96.2)	(20)	(0.31–0.80)	(95.9)	(25)	(0.42–0.97)	(95.6)	(21)	(0.34–0.84)	(95.1)	(24)	(0.42–0.97)
		20–29	5383	5,383	0.00	3,193	3,193	6.26	14,126	14,126	4.25	15,282	15,282	3.93	16,296	16,296	3.68	17,650	17,650	3.40
			(1.5)	(0)	(0.00–0.00)	(0.9)	(2)	(0.0–14.94)	(3.8)	(6)	(0.85–7.65)	(4.1)	(6)	(0.79–7.07)	(4.4)	(6)	(0.74–6.63)	(4.9)	(6)	(0.68–6.12)
		30≤	58	58	0.00	26	26	0.00	50	50	0.00	53	53	0.00	54	54	0.00	67	67	0.00
			(0)	(0)	(0.00–0.00)	(0.0)	(0)	(0.00–0.00)	(0.0)	(0)	(0.00–0.00)	(0.0)	(0)	(0.00–0.00)	(0.0)	(0)	(0.00–0.00)	(0.0)	(0)	(0.00–0.00)

HIV: human immunodeficiency virus, Tests (N): The number of HIV tests, Persons (N): The number of HIV test-takers, HIV+: The number of HIV-infected persons, Pre: HIV Prevalence (the number of HIV-infected persons per 10,000 HIV test-takers, BB: blood bank, PHCs: public health centers, MMA: The Military Manpower Administration, CI: confidence interval.

Sex and age distributions of HIV test-takers showed different characteristics among the 3 testing institutes. Among blood donors, approximately 80% of the HIV test takers were adolescents and young adults, 70% were men, and sex distribution was similar across the years. For PHC visitors, approximately 70% were women, and 40% were adolescents and young adults. Finally, 100% of test-takers at MMA were male, and 95–99% of them were teenagers.

**Figure 2:**
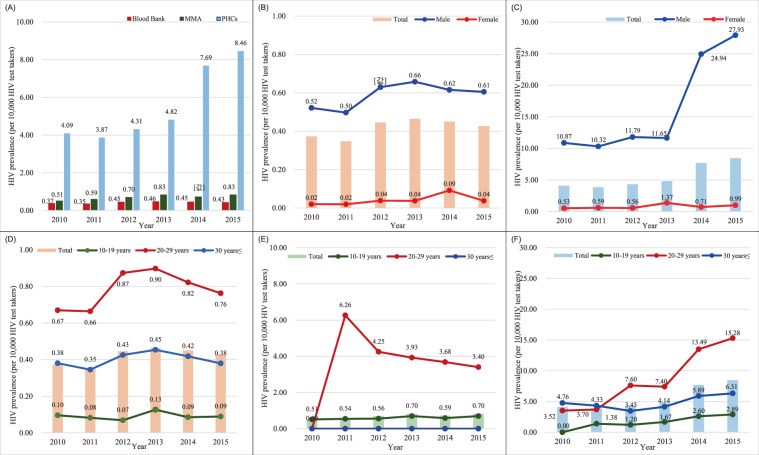
Comparison of status of HIV prevalence distribution by sex, age, and the institution at which the initial HIV diagnosis was performed, in Korea, from 2010 to 2015. (**a**) Distribution of HIV prevalence by HIV screening institution in which the initial diagnosis was performed, in Korea, from 2010 to 2015; MMA: the Military Manpower Administration, PHCs; public health centers. (**b**) Trends in HIV prevalence by sex, at blood banks, from 2010 to 2015. (**c**) Trends in HIV prevalence by sex, in public health centres, from 2010 to 2015. (**d**) Trends in HIV prevalence by age, at blood banks, from 2010 to 2015. (**e**) Trends in HIV prevalence by age, according to the Military Manpower Administration, from 2010 to 2015. (**f**) Trends in HIV prevalence by age, at public health centres, from 2010 to 2015.

HIV prevalence across the three testing institutes almost doubled during the study period from 0.89 per 10,000 test-takers in 2010 to 1.68 in 2015. HIV prevalence was higher in males than in females. In males, the prevalence was 1.19 per 10,000 test-takers in 2010, and it increased every year up to 2.33 in 2015 (*p* < 0.0001). In females, the prevalence was 0.17 in 2010 and 0.35 in 2015, showing an irregularly increasing trend for six years. By age group, HIV prevalence in 2010 was 2.10 in adults aged 30 years or older, 1.01 in young adults aged 20–29 years, and 0.23 in adolescents aged 10–19 years. HIV prevalence increased in all the age groups in 2015, with 3.01, 2.45, and 0.34, respectively. Of the three age groups, young adults showed the most considerable change, as the HIV prevalence doubled during the six years. Regarding HIV prevalence by testing institutes, the prevalence in 2010 was 4.09 per 10,000 test-takers in PHCs, 0.51 in MMA and 0.37 in BB; in 2015 it was 8.46 in PHCs, 0.83 in MMA, and 0.43 in BBs, all showing an increase (*p* < 0.0001) [Fig. 2a]. HIV prevalence among visitors to PHCs showed the most considerable change, increasing by more than two times. Among both blood donors and visitors to PHCs, the prevalence was higher in males, and especially young adults [Fig. 2b–d,f]. Among males who underwent a physical examination at MMA, HIV prevalence in adolescents was 0.51–0.70 [Fig. 2e].

### The characteristics of HIV prevalence in young male adults by the reason for HIV testing

For the reason for HIV testing, we analyzed data of young male adults aged 20–29 years showing the highest HIV prevalence across the 3 testing institutes and the most considerable change in annual HIV prevalence during the study period. Since 2013, the number of blood donors in young male adults increased to more than 500,000. However, HIV prevalence did not significantly change, with a range of 0.91–1.19 per 10,000 test-takers (*p* = 0.4765). Most males at MMA were teenagers, and the number of young male adults was under 6,000 until 2012 but increased to over 14,000 since then. HIV prevalence among them was the highest in 2011 (6.37). However, it decreased every year since then, to 3.40 in 2015. There were changes in the number of HIV testing among PHC visitors according to the reason for taking the HIV test. The number of individuals tested as part of a health check-up or prenatal check-up increased, while the figure among tuberculosis patients, prisoners, and individuals with a high risk of STI decreased. HIV prevalence among PHC visitors according to the reason for HIV testing was the highest in those with a suspected HIV infection, followed by those referred by a doctor or visited a PHC for a health check-up. The prevalence significantly increased annually. Mainly, among PHC visitors, HIV prevalence in young male adults with a suspected HIV infection increased by 6.5 times in 2014 and 2015 (315.79 in 2014 and 335.55 in 2015) (p < 0.0001), compared to before 2014, although the annual number of such individuals did not increase (*p* < 0.0001). Further, the HIV prevalence of young male adults referred by a doctor increased by 14.3 times during the same period in PHCs (9.51 in 2010, 135.48 in 2014, and 100.00 in 2015) (*p* < 0.0001) [Table 2].

**Table 2:** Change in HIV prevalence among young male adults (20–29 years) by the reason for HIV testing at HIV screening institutes in Korea from 2010 to 2015.

Reason for HIV testing	2010			2011			2012			2013			2014			2015		
	Persons(N) (HIV+)	Pre (95% CI)	aOR (95% CI)	Persons(N) (HIV+)	Pre (95% CI)	aOR (95% CI)	Persons(N) (HIV+)	Pre (95% CI)	aOR (95% CI)	Persons(N) (HIV+)	Pre (95% CI)	aOR (95% CI)	Persons(N) (HIV+)	Pre (95% CI)	aOR (95% CI)	Persons(N) (HIV+)	Pre (95% CI)	aOR (95% CI)
Blood dona. in BB	495,004	0.91	1	444,483	0.92	1	469,386	1.19	1	510,677	1.19	1	535,238	1.06	1	526,932	1.01	1
	(45)	(0.64–1.17)		(41)	(0.64–1.20)		(56)	(0.88–1.51)		(61)	(0.89–1.49)		(57)	(0.79–1.34)		(53)	(0.74–1.28)	
Conscript in MMA	5,383	0.00	—	3,139	6.37	6.63	14,126	4.25	3.52	15,222	3.94	3.25	16,296	3.68	3.30	17,650	3.40	3.36
	(0)	(0.00–0.00)		(2)	(0.00–15.20)	(1.60–27.44)	(6)	(0.85–7.65)	(1.52–8.18)	(6)	(0.79–7.10)	(1.41–7.54)	(6)	(0.74–6.63)	(1.42–7.66)	(6)	(0.68–6.12)	(1.45–7.83)
Health check-up	3,241	15.4	24.58	5,107	7.83	8.84	6,033	4.97	4.53	6,710	5.96	5.29	5,605	7.14	7.25	5,716	10.50	12.12
	(5)	(1.92–28.94)	(9.65–62.31)	(4)	(0.16–15.51)	(3.16–24.70)	(3)	(0.00–10.60)	(1.42–14.48)	(4)	(0.12–11.80)	(1.92–14.55)	(4)	(0.15–14.13)	(2.63–20.01)	(6)	(2.10–18.89)	(5.20–28.25)
Medical certificate	4,709	0.00	—	3,455	0.00	—	6,911	4.34	3.95	5,579	0.00	—	3,945	7.60	7.73	3,525	14.18	16.38
	(0)	(0.00–0.00)		(0)	(0.00–0.00)		(3)	(0.00–9.25)	(1.24–12.64)	(0)	(0.00–0.00)		(3)	(0.00–16.21)	(2.42–24.71)	(5)	(1.76–26.61)	(6.54–41.07)
Prenatal checkup	2,847	0.00	—	7,006	0.00	—	4,498	0.00	—	7,479	0.00	—	8,241	0.00	—	9,310	0.00	—
	(0)	(0.00–0.00)		(0)	(0.00–0.00)		(0)	(0.00–0.00)		(0)	(0.00–0.00)		(0)	(0.00–0.00)		(0)	(0.00–0.00)	
Referral by doctor	2,102	9.51	15.11	2,234	22.38	24.85	1,947	77.04	70.77	1,825	60.27	53.71	1,550	135.48	139.51	1,500	100.00	116.65
	(2)	(0.00–22.70)	(3.64–62.66)	(5)	(2.79–41.98)	(9.80–62.97)	(15)	(38.20–115.88)	(39.89–125.54)	(11)	(24.76–95.79)	(28.22–102.47)	(21)	(77.93–193.04)	(84.13–231.35)	(15)	(49.65–150.35)	(65.43–207.95)
Suspected	3,874	51.63	82.35	4,356	66.57	74.24	5,252	68.55	62.90	5,343	78.61	70.27	2,850	315.79	331.48	3,308	335.55	402.32
	(20)	(29.0–74.19)	(47.90–141.59)	(29)	(42.42–90.72)	(46.02–119.76)	(36)	(46.23–90.86)	(41.26–95.88)	(42)	(54.93–102.29)	(47.30–104.37)	(90)	(251.59–379.99)	(236.31–464.98)	(111)	(274.18–396.92)	(288.12–561.79)
TB Patient	574	0.00	-	326	0.00	—	657	0.00	—	333	0.00	—	294	0.00	—	207	0.00	—
	(0)	(0.00–0.00)		(0)	(0.00–0.00)		(0)	(0.00–0.00)		(0)	(0.00–0.00)		(0)	(0.00–0.00)		(0)	(0.00–0.00)	
Prisoner	5,730	0.00	—	924	0.00	—	2,321	12.93	11.78	1,286	7.78	6.90	742	13.48	13.70	697	14.35	16.57
	(0)	(0.00–0.00)		(0)	(0.00–0.00)		(3)	(0.00–27.54)	(3.68–37.70)	(1)	(0.00–23.01)	(0.96–49.82)	(1)	(0.00–39.87)	(1.89–99.11)	(1)	(0.00–42.45)	(2.29–120.19)
STI risk group	8,479	0.00	—	8,869	0.00	—	7,8141	0.00	—	5,812	1.72	1.53	3,571	2.80	2.84	3,005	0.00	—
	(0)	(0.00–0.00)		(0)	(0.00–0.00)		(0)	(0.00–0.00)		(1)	(0.00–5.09)	(0.21–11.01)	(1)	(0.00–8.29)	(0.39–20.55)	(0)	(0.00–0.00)	

N: the number of HIV test-takers, (HIV+): the number of HIV-infected persons, Pre: HIV Prevalence (the number of HIV-infected persons per 10,000 HIV test-takers), CI: confidence interval, aOR: Adjusted (nationality, sex, region) Odds Ratio, Blood dona. in BB: Blood donation tested in Blood Bank, Conscript in MMA: conscript tested in the Military Manpower Administration, health check-up, medical certificate, prenatal check-up, referral by doctor, suspected, TB patient, prisoner, and STI risk group tested in the public health center, STI: Sexual Transmission Infection.

## Discussion

This study investigated the sizes of HIV testing and the characteristics of HIV-infected adolescents and young adults in Korea using data at BBs, PHCs, and MMA that frequently perform HIV tests on adolescents and young adults. HIV-infected teenagers at the testing institutes accounted for 96% of those in the same age group identified as those HIV-infected in Korea overall, and the corresponding rate for individuals in their 20 s was 50%. Therefore, the monitoring of HIV infection in BBs, PHCs, and MMA was found to be a suitable HIV surveillance method for adolescents and young adults in Korea. Furthermore, the institutes showed distinctive features. HIV-infected young adults among PHC visitors constituted approximately 30% of all HIV-infected individuals nationwide in the same age group, while HIV-infected teenaged males at the MMA comprised approximately 50% of HIV-infected individuals nationwide in the same age group.

It has been argued that the increase in the total number of HIV-infected individuals in Korea is due to an increase in HIV infection among adolescents and young adults in recent years. According to a KCDC report, in 2006, individuals newly diagnosed with HIV were mainly in their 30 s (29.4%) and 40 s (24.3%). However, since 2012 the age group with the most significant number of HIV-infected individuals shifted to the 20 s (30.4%), followed by the 30 s (23.3%). In 2015, rates in young adults further increased, constituting as much as 33.2% of all newly identified cases of HIV. We identified the characteristics of the increase in new diagnoses of HIV among these age groups, by analyzing the changes in the rate of HIV tests taken and HIV prevalence by the reason for HIV testing, using the data from BB, PHCs, and MMA. Neither the number of HIV tests undertaken by teens across the three testing institutes changed over the six years (*p* = 0.1369), nor did the HIV prevalence (0.23 in 2010, 0.34 in 2015) (*p* = 0.5746). Therefore, it cannot be explicitly concluded that HIV infection increased in Korean teens. Every year, MMA performs HIV tests in approximately 350,000 males and has identified half of the total HIV-infected teens, the results of which can be used as a critical national-level HIV monitoring index to represent the current status of HIV infection among Korean teens. However, concerning young adults, the rate of HIV tests conducted increased by approximately 200,000 cases over the six years, and HIV prevalence increased by more than two-fold as well (1.01 in 2010, 2.45 in 2015). Notably, for young male adults, the HIV prevalence in the “suspected for HIV infection” group, in PHCs increased rapidly (over 300 in 2014 and 2015). A large number of HIV-infected individuals were identified among PHC visitors since 2014, which was reflective of the rapid increase in HIV prevalence since 2014. It was suspected that this was due to actively introducing and expanding various opportunities for HIV testing, including rapid diagnostic tests at PHCs in Seoul since 2014^14,15^. Over the past ten years, STI prevalence in Korea has been increasing among individuals in their 30 s and older^16^. However, this trend has changed, with the affected age group is now younger^17^.

In our study, the increase in HIV infection among young adults was identified in males only. According to a report on the HIV status in Korea, the male to female ratio in the patient population was increased continuously from 10.2:1 in 2010 to 15.0:1 in 2015^3^. Also, a study on mode of HIV transmission in Korea predicted that the increase in young male patients in Korea was caused by escalating male-male sexual contacts^18^. Therefore, young male adults, in particular, should have access to specific education to promote HIV prevention.

According to the World Health Organization (WHO), in 2013, young people aged between 15 and 24 years were estimated to constitute 35% of newly diagnosed cases of HIV worldwide^19^. The HIV infection prevalence among adolescents and young adults in all of Japan, Taiwan, the US, and Europe needs our attention. In Japan, HIV infection among young people has increased since 2000, and particularly in 2016, 33.4% of all newly diagnosed HIV patients between 15 and 29 years old^20^. In Taiwan, individuals between 10 and 29 years old constituted 47.8% of all newly diagnosed cases^21^. In the US, recent reports suggest that HIV infection was the highest among 25- to 29-year-olds, and the infection rate in this age group is gradually increasing. Particularly, 41.4% of those identified as HIV-infected up until 2015 were between 13 and 29 years^22^. In European countries as well, 20–29 years were identified as the age group with the highest number of HIV-infected individuals^23^. Thus, a higher prevalence of HIV infection in young people emerges as a global pattern. Consequently, these studies demonstrate the importance of identifying significant groups responsible for HIV transmission and establishing HIV prevention strategies appropriate to each transmission group.

In Korea, sexual contact is the primary route of HIV transmission and prevalence, yet mortality rates have so far been low relative to other countries^10^. Further, to prevent HIV transmission, the Korean government provides proactive and continuous treatment support to a vast majority of registered HIV-infected individuals (approximately 94%). However, this support is not available to those who test anonymously—those who do not wish to expose their personal information. To date, 15,579 Korean have registered in the HIV database, and the government continuously supports treatments for the current survivors (n = 12,991) as of 2018.

Once referred to a hospital for treatment, the government pays all costs associated with testing and treatment. With that, the survival of HIV-infected individuals has been prolonged, and the demand for various healthcare/long-term care services has increased due to an increase in old-age related chronic illnesses. HIV requires lifelong treatment as presently, there is no cure. Thus, HIV-infection in young adults can place a further economic and societal burden of the disease on the nation as well as the individual.

Concerning the reason for HIV testing among individuals recently identified as HIV-infected in Korea, HIV testing due to suspected HIV infection was on an increasing trend^15^. In the past, most HIV infection patients were diagnosed at hospitals and clinics, but the number of HIV-infected individuals diagnosed at PHCs has increased recently. In Korea, education, health promotion, and counseling are provided to prevent HIV transmission via sexual contact. Specifically, KCDC fosters knowledge-based and behavioral changes through education in the format of musicals (targeting adolescents), promotional material contests (targeting college students), and Naver Webtoon a weekly online cartoon series that addresses AIDS prevention (targeting people in their 20 s and 30 s).

## Conclusion

It was found that the recent overall increase in HIV infection among Koreans was due to an increase in HIV infection in young adults rather than in adolescents. Specifically, we conclude that the growing HIV infection rate in Korea is due to the increased HIV prevalence among suspected young adults at PHCs. Accordingly, we suggest specific and aggressive HIV prevention programs for adolescents before they reach the age of 20, and their exposure to sex increases. Another finding of the study was that HIV prevalence is increasing among young male adults. HIV prevalence in males undergoing physical examination at MMA can function as the best index for the status of HIV infection among Korean teens.
