# Author Correction: Characteristics of Adolescents and Young adults with HIV in the Republic of Korea from 2010 through 2015

**DOI:** 10.1038/s41598-020-70098-8

**Published:** 2020-08-04

**Authors:** Myeongsu Yoo, Jaehyun Seong, Jae-Gyu Yoon, Jeong-ok Cha, Yoon-Seok Chung, Kisoon Kim, Mee-Kyung Kee

**Affiliations:** 1 Division of Viral Disease Research, Center for Infectious Disease Research, Korea National Institute of Health, Chungbuk, Republic of Korea; 2 Division of TB and HIV/AIDS Control, Center for Disease Prevention, Korea Center for Disease Control & Prevention, Chungbuk, Republic of Korea; 3 Division of Viral Disease, Center for Laboratory Control of Infectious Disease, Korea Center for Disease Control and Prevention, Chungbuk, Republic of Korea

Correction to: *Scientific Reports*https://doi.org/10.1038/s41598-020-66314-0, published online 10 June 2020

The original version of this Article contained errors.

In the Abstract,

“In the three testing institutes, there were new cases of HIV with 50% and 95% of cases diagnosed in young adults and adolescents, respectively.”

now reads:

“In the three testing institutes, there were new cases of HIV with 50% and 75% of cases diagnosed in young adults and adolescents, respectively.”

In the Results section, under the subheading ‘The status and trends of HIV infection by age and testing institute’,

“Adolescents aged 10–19 years identified at the 3 institutes constituted 96% of the total number of HIV-infected individuals in the same age range; young adults aged 20–29 years constituted more than 50% of the total number of HIV infections in the corresponding age range.”

now reads:

“Adolescents aged 10–19 years identified at the 3 institutes constituted 75% of the total number of HIV-infected individuals in the same age range; young adults aged 20–29 years constituted more than 50% of the total number of HIV infections in the corresponding age range.”

and,

“The proportions were approximately 18% and 28% in the case of blood donors and PHC visitors, respectively [Fig. 1].”

now reads:

“The proportions were approximately 16% and 8% in the case of blood donors and PHC visitors, respectively [Fig. 1].”

In the Discussion,

“HIV-infected teenagers at the testing institutes accounted for 96% of those in the same age group identified as those HIV-infected in Korea overall, and the corresponding rate for individuals in their 20 s was 50%.”

now reads:

“HIV-infected teenagers at the testing institutes accounted for 75% of those in the same age group identified as those HIV-infected in Korea overall, and the corresponding rate for individuals in their 20 s was 50%.”

These errors have now been corrected in the PDF and HTML versions of the Article.

